# An Explanation of Why Dose-Corrected Area Under the Curve for Alternate Administration Routes Can Be Greater than for Intravenous Dosing

**DOI:** 10.1208/s12248-024-00887-w

**Published:** 2024-01-30

**Authors:** Hirokazu Wakuda, Yue Xiang, Jasleen K. Sodhi, Naoto Uemura, Leslie Z. Benet

**Affiliations:** 1 Department of Bioengineering and Therapeutic Sciences, Schools of Pharmacy and Medicine, University of California, San Francisco, San Francisco, California 94143-0912, USA; 2 Department of Clinical Pharmacology and Therapeutics, School of Medicine, Oita University, 1-1 Idai gaoka, Hasama-machi, Yufu City, Oita 879-5593, Japan; 3 Department of Drug Metabolism and Pharmacokinetics, Septerna, South San Francisco, California 94080, USA

**Keywords:** bioavailability, Kirchhoff’s Laws, systemic concentrations, urinary excretion

## Abstract

It is generally believed that bioavailability (F) calculated based on systemic concentration area under the curve (AUC) measurements cannot exceed 1.0, yet some published studies report this inconsistency. We teach and believe, based on differential equation derivations, that rate of absorption has no influence on measured systemic clearance following an oral dose, i.e., determined as available dose divided by AUC. Previously, it was thought that any difference in calculating F from urine data *versus* that from systemic concentration AUC data was due to the inability to accurately measure urine data. A PubMed literature search for drugs exhibiting F>1.0 and studies for which F was measured using both AUC and urinary excretion dose-corrected analyses yielded data for 35 drugs. We show and explain, using Kirchhoff’s Laws, that these universally held concepts concerning bioavailability may not be valid in all situations. Bioavailability, determined using systemic concentration measurements, for many drugs may be overestimated since AUC reflects not only systemic elimination but also absorption rate characteristics, which is most easily seen for renal clearance measures. Clearance of drug from the absorption site must be significantly greater than clearance following an iv bolus dose for F(AUC) to correctly correspond with F(urine). The primary purpose of this paper is to demonstrate that studies resulting in F>1.0 and/or greater systemic *vs* urine bioavailability predictions may be accurate. Importantly, these explications have no significant impact on current regulatory guidance for bioequivalence testing, nor on the use of exposure (AUC) measures in making drug dosing decisions.

## Introduction

In Wagner’s 1981 comprehensive review of the “History of Pharmacokinetics” (1), it appears that analysis of concentration-time curves following oral dosing and considerations of bioavailability began in the 1940s. Those early analyses and all subsequent analyses until today have derived the concepts in terms of amounts and rate constants utilizing differential equations, which is appropriate in the field of Chemistry. But since following human dosing we measure drug concentrations and define elimination processes in terms of clearance values, the differential equation derivations and their solutions are divided by a volume of distribution. Then, it is possible to define the clearance by measuring the amount eliminated divided by the exposure driving that elimination, as we recently reviewed (2). Our laboratory has emphasized the possibility that the approach followed in chemistry, in terms of rate constants and differential equations for disposition processes in a fixed volume of fluid, may not be consistent with the pharmacokinetic, clearance and varying volumes of distribution, approach (3, 4). A number of peer-reviewed published manuscripts report drug bioavailabilities for differing routes of drug dosing compared to intravenous bolus doses that exceed 100%. In Table I, we cite thirty-four such outcomes following crossover studies in which humans and various animal species received a drug both by intravenous dosing and via either oral, subcutaneous (SubQ), or intramuscular (IM) dosing, where the reported ratio of dose-corrected areas under the systemic concentration-time curves (${\text{AUC}}_{x}∕{\text{AUC}}_{\text{iv}}$) exceeded 1.02. Sixteen of these thirty-four studies were in humans.

Another series of unexplained data pertains to the differences observed in dose-corrected oral bioavailability when comparing measures of AUC to measures of the amount of drug excreted unchanged in the urine. Eleven such peer-reviewed human studies are referenced in Table II, including three of the studies in Table I. Since the measured outcomes in Tables I and II are not consistent with the universally accepted or FDA guidance methods for calculating bioavailability, these data were generally ignored and believed to result from experimental errors. We will revisit these data in the “Discussion” section.

## Methods and Results

### Literature Search

The literature search for the data listed in Tables I and II was a very laborious task as we were searching for published results that are contrary to generally accepted pharmacokinetic theory, as bioavailability measurements greater than unity have typically been attributed to some form of experimental error. Information as to this outcome is rarely, if ever, included in article titles or abstracts. In essence, we searched for any peer-reviewed bioavailability study in humans and animals. Specifically, we focused on studies where oral, SubQ, or IM dosing was reported alongside iv dosing to determine if the published data had values of dose-corrected AUC greater than 1.0 and/or where F calculated using urinary measurements was lower than that obtained through systemic concentration measurements. We put no period of time restriction on studies to evaluate and as can be seen, the cited references range from 1976 to 2022. The only relevant search term was “bioavailability,” but as can be seen in the 33 citations in Tables I and II, one-third were not found using this search term. It should not be concluded that since we found so few studies meeting our criteria this means the phenomena occurs to a negligible extent, since investigators both in academia and the industry are reluctant to publish results for bioavailability studies that are contrary to accepted theory and thus will be believed by editors, journal reviewers, and readers, and even the investigators themselves, to be scientifically flawed.

### Application of Kirchhoff’s Laws to Eliminate the Need for Solving Differential Equations

We recently demonstrated (4), adapting Kirchhoff’s Laws from physics, that overall rate constants for a linear kinetic process or overall clearance for that process can be directly derived without the need to use differential equations. As we first reported (3), the application of Kirchhoff’s Laws to clearance can be summarized in Eq. 1 for parallel processes and Eq. 2 for processes in series.


(1) $$CL_{total}=CL_{rate\;defining\;parallel\;process\;1}+CL_{rate\;defining\;parallel\;process\;2}+\ldots$$

(2) $$\frac{1}{CL_{total}}=\frac{1}{CL_{rate\;defining\;in\;series\;process\;1}}\;\;+\frac{1}{CL_{rate\;defining\;in\;series\;process\;2}}+\cdots$$

Kirchhoff’s Laws may also be applied to rate constants and can be derived via Eqs. 3 and 4, independent of solving differential equations for first-order processes.


(3) $$k_{total}=k_{rate\;defining\;parallel\;proces\;1}+k_{rate\;defining\;parallel\;proces\;2}+\ldots$$

(4) $$\frac{1}{k_{total}}=\frac{1}{k_{rate\;defining\;in\;series\;process\;1}}+\frac{1}{k_{rate\;defining\;in\;series\;process\;2}}+\cdots$$

A rate-defining process is defined by a parameter that describes an elimination or movement process for which it is possible under certain conditions that the total clearance or total rate constant may be equal to this parameter. For example, a rate-defining clearance process for hepatic elimination could be hepatic blood flow, i.e., the rate at which the drug arrives to the liver is the maximum value that hepatic elimination can be. Thus, for a very high hepatic clearance ($CL_{H}$) drug, total $CL_{H}$ could equal hepatic blood flow ($Q_{H}$). To exemplify a rate-defining rate constant process, for a series of chemical reactions occurring in a beaker, the elimination rate constant for the parent drug could be the minimum value rate-defining process for all subsequent metabolic steps. For example, if the first step in a metabolic elimination process is very slow, the observed rate constant for the subsequent metabolic steps will be that initial rate constant for the metabolism of the parent drug. Understanding this definition is essential in applying Kirchhoff’s Laws. As our approach is contrary to accepted methodology in pharmacokinetics utilizing differential equations, we expected and have seen recent publications challenging the validity of our methodology (38, 39). We address these differences in “Discussion.” The critical aspect of our approach is that only rate-defining processes can be combined to determine the overall rate constant for elimination or clearance following Kirchhoff’s Laws. Passive permeability, no matter how slow, cannot be a rate-defining process for elimination because passive permeability is reversible, i.e., clearance and elimination rate will never be equal to passive permeability. When hepatic basolateral transporters affect permeability and active influx is greater than active efflux, this can be a rate-defining process. But not when active efflux is greater than active influx. That is, clearance can never be defined singly as active large efflux minus smaller active influx since the value is negative.

Examples of parallel rate-defining processes in the kidney are glomerular filtration and secretion/reabsorption, and in the liver are metabolism and biliary excretion (3). Examples of in-series rate-defining processes in the kidney and liver are organ blood flow limiting the rate of elimination, so that the actually eliminating mechanism has no effect on the measured rate (4). Of particular relevance in this publication are the in-series processes of absorption and elimination, where absorption rate or absorption clearance from the gut (as well as from an injection site whether it is IM or SubQ) can have an effect on the overall elimination process.

### Kirchhoff’s Laws Derivations of Overall Rate of Elimination and Overall Clearance Following Oral Dosing

#### Rate Constant Derivation

Kirchhoff’s Laws may be used to calculate the overall rate of elimination for in-series absorption-elimination processes.


(5) $$\frac{1}{k_{overall\;measured\;rate}}=\frac{1}{k_{entering}}+\frac{1}{k_{leaving}}=\frac{1}{k_{a}}+\frac{1}{k_{iv\;bolus\;dose}}$$

Although Eq. 5 may appear to be new to pharmacokineticists, in fact as we reported (4), we have been using and accepting its equivalent for more than 40 years since Yamaoka *et al*. (40) recognized that the absorption-disposition model could be described by mean residence time (*MRT*) concepts:

(5a) $$MRT_{measured\;following\;oral\;dosing}=MAT+MRT_{measured\;following\;iv\;bolus\;dosing}$$

where MAT is the mean absorption time. Since the inverse of the mean residence time for each of the processes is the first-order rate constant characterizing the process, $k_{a}$ is the absorption rate constant and $k_{\text{iv bolus dose}}$ is the single rate constant characterizing elimination for a one-compartment or a multicompartment body model following an iv bolus dose. Solving Eq. 5 gives

(6) $$k_{overall\;measured\;rate}=\frac{k_{a}\cdot k_{iv\;bolus}}{k_{a}+k_{iv\;bolus}}=\frac{k_{iv\;bolus}}{1+\frac{k_{iv\;bolus}}{k_{a}}}$$

It is well recognized that for flip-flop models (41), absorption may be so slow that it becomes the rate-limiting process and the terminal disposition constant for the concentration-time curve following oral dosing is $k_{a}$. Equation 6 explains this (i.e., if $k_{a}$ is very small compared to $k_{\text{iv bolus}}$, the value of 1 in the denominator becomes negligible and the $k_{\text{iv bolus}}$ terms cancel) and provides the basis for the overall measured rate constant for intermediate values.

#### Clearance Derivation

As we recently presented (4), in-series absorption clearance processes can also be derived using Kirchhoff’s Laws in terms of clearance entering into the systemic circulation and clearance leaving from the systemic circulation

(7) $$\frac{1}{CL_{after\;oral\;dosing}}=\frac{1}{CL_{entering}}+\frac{1}{CL_{leaving}}=\frac{1}{CL_{gut}}+\frac{1}{CL_{iv\;dosing}}$$

where the $CL_{\text{entering}}$ is the clearance of the drug from the gut, a parameter previously unmeasured in pharmacokinetics, but can be simply envisioned as the absorption rate constant, $k_{a}$, multiplied by the volume of distribution of the gut compartment, again a parameter previously unmeasured in pharmacokinetics. Since overall clearance is defined as the amount eliminated from the systemic fluids (bioavailability, F, multiplied by the administered dose, ${\text{Dose}}_{\text{oral}}$), divided by the area under the systemic concentration-time curve over all time $\left(AUC_{0\to \infty }\right)$, solving Eq. 7 yields

(8) $$CL_{after\;oral\;dosing}=\frac{F\cdot Dose_{oral}}{AUC_{0\to \infty }}=\frac{CL_{gut}\cdot CL_{iv\;dose}}{CL_{gut}+CL_{iv\;dose}}=\frac{CL_{iv\;dose}}{1+\frac{CL_{iv\;dose}}{CL_{gut}}}$$

There are important implications to Eq. 8. First, Eq. 8 demonstrates that the clearance measured after oral dosing will not be the clearance after intravenous dosing unless ${\text{CL}}_{\text{gut}}\gg {\text{CL}}_{\text{iv dose}}$, which may often hold true but just as likely not true. Equation 8 is completely new to pharmacokinetics and thus can elicit strong resistance to its acceptance. However, to argue against the validity of Eq. 8, one must maintain that while it is possible to have elimination be rate-limited by absorption, i.e., flip-flop pharmacokinetics, it is implausible for overall clearance to be rate-limited by gut clearance. Yet, this is what our field has been maintaining (i.e., absorption processes have no effect on the measured AUC) ever since the importance of clearance concepts was recognized, as we demonstrate in the next section.

Although Eq. 8 is completely new to pharmacokinetics in terms of gut absorption, the concept that a slow clearance early in a series of metabolic steps will affect clearance measures later in the process is obvious. Consider our earlier publication (4) where we applied Kirchhoff’s Laws to determine rate constants for in-series metabolic steps (Drug to Metabolite 1 to Metabolite 2 to Metabolite 3). If the measured clearance of Drug to Metabolite 1 is very slow, the measured clearances of Metabolite 1 to Metabolite 2 and Metabolite 2 to Metabolite 3 in this study cannot be faster than the measured clearance of Drug to Metabolite 1.

The second important implication to Eq. 8 is that an experimental methodology is available to determine if slow clearance of drug from the gut (or any absorption site including IM, SubQ, oral controlled release dosage forms, and even prolonged zero order iv infusions) affects AUC. That methodology is a measurement of renal clearance, as we will also address subsequently. If an input process causes an increase in AUC relative to the same iv bolus dose, renal clearance will also decrease for an alternative route of administration relative to the iv bolus.

The third important implication to Eq. 8 is that more than one volume of distribution is implicit in Eq. 8 (i.e., systemic volume of distribution and gut volume of distribution). Both volume terms must be used to convert Eq. 6 into Eq. 8. Thus, it is possible to understand why the traditional chemistry approach to deriving rates of elimination using differential equations may not be correct for pharmacokinetic derivations since only one volume term can be inserted into the chemistry differential equation derivation (4).

### The Error in the Use of a Differential Equation Derivation to Determine Systemic Clearance Following Oral Absorption

Since this is such an important aspect in understanding the data in Tables I and II, we repeat the common derivation followed for the past 50 years for simplicity for the 1-compartment body model where $k_{\text{iv bolus}}=k_{d}$ (4). The amount of drug in the systemic circulation as a function of time in terms of the rate constants utilized above is given by Eq. 9.


(9) $$A_{systemic\;circulation}=\frac{k_{a}\cdot F\cdot Dose_{oral}}{k_{a}-k_{d}}\cdot (e^{-k_{d}\cdot t}-e^{-k_{a}\cdot t})$$

Note that in the derivation of Eq. 9, it is assumed that only the available dose (F⋅Dose) drives absorption from the gut (i.e., $\frac{dA_{gut}}{dt}=-k_{a}\cdot F\cdot Dose$). Why should this be true? This assumes that drug metabolized upon first pass through the gut tissue and the liver has no effect on the rate of drug absorption even though these metabolic processes take place after absorption has occurred. But this assumption is generally accepted without question, and not even mentioned until we raised the issue.

Secondly, the correct differential equation for loss of drug from the gut is actually $\frac{dA_{gut}}{dt}=-k_{gut}\cdot Dose$, where $k_{\text{gut}}$ is the sum of $k_{a}$ (which itself is a hybrid parameter summarizing multiple sequential processes, in the case of solid oral dosage forms encompassing gastrointestinal transit, disintegration, dissolution, and diffusion) and any other elimination process in the gut, such as degradation. Thus, the correct relationship is

(9a) $$A_{systemic\;circulation}=\frac{k_{a}\cdot F\cdot Dose_{oral}}{k_{gut}-k_{d}}\cdot (e^{-k_{d}\cdot t}-e^{-k_{gut}\cdot t})$$

which the field also generally ignores.

Dividing Eq. 9 by the systemic volume of distribution (V) gives the concentration–time relationship

(10) $$C_{systemic\;circulation}=\frac{k_{a}\cdot F\cdot Dose_{oral}}{\left(k_{a}-k_{d}\right)\cdot V}\cdot (e^{-k_{d}\cdot t}-e^{-k_{a}\cdot t})$$

Then integrating over all time allows determination of $AUC_{0\to \infty }$

(11) $$AUC_{0\to \infty }=\frac{\frac{k_{a}\cdot F\cdot Dose_{oral}}{(k_{a}-k_{d})\cdot V}}{k_{d}}-\frac{\frac{k_{a}\cdot F\cdot Dose_{oral}}{(k_{a}-k_{d})\cdot V}}{k_{a}}=\frac{\frac{k_{a}\cdot F\cdot Dose_{oral}}{V}}{k_{a}\cdot k_{d}}=\frac{F\cdot Dose_{oral}}{k_{d}\cdot V}$$

(12) $$\text{Therefore},\;CL_{after\;oral\;dosing\;(differential\;equations)}=\frac{F\cdot Dose_{oral}}{AUC_{0\to \infty }}\;\;=k_{d}\cdot V=CL_{iv\;bolus}$$

where our field implicitly believes a slow absorption rate can markedly affect the overall rate of elimination (Eq. 6), but it does not affect the overall clearance of elimination (Eq. 12). Note that Eq. 12 will be derived no matter how many exponentials are required to characterize the iv bolus dose equation or the equation describing loss from the gut compartment, where the first order rate constant for elimination from the measured compartment multiplied by the volume of distribution of the measured compartment will equal $CL_{iv\;\text{bolus}}$ for linear first-order processes. We do recognize that prior to examination of the Kirchhoff’s Laws derivations for clearance and rate constants, only differential equation derivations were possible, thus requiring ignoring any potential difference in volumes of distribution that influence differing processes.

### Measurements of Renal Clearance Following Oral and Intravenous Dosing

It is generally believed today that renal clearance will be unchanged following oral and intravenous dosing, just as it is presently believed that input rate following oral, IM, and SubQ dosing will have no effect on the area under the systemic concentration-time curve. The advantage of examining this renal clearance belief is that the calculation of $CL_{R}$ is not based on any assumptions (2), just that renal clearance is equal to the measured amount of drug eliminated unchanged in the urine divided by the measured systemic exposure driving that elimination ($AUC_{0\to \infty }$). In contrast, *F* in Eqs. 9–12 is not a measured value, but rather a calculated value. Thus, if input does increase the systemic concentration–time curve, then the renal clearance calculated following oral, IM, and SubQ dosing should be less than the renal clearance following iv dosing.

Such analyses, not previously considered, are possible for the 1-deamino-8-arginine vasopressin (6), sodium fluoride (18), and cimetidine (21) studies. For each of the 16 cimetidine oral dosings in 9 subjects (21), it is possible to compare these renal clearances, which average 21.6 ± 10.6 L/h following oral dosing and 35.6 ± 10.0 L/h following iv dosing (paired *t*-test for the oral – iv difference yielded a *p* value of 0.0018). When renal clearances of the 10 paired dosings of sodium fluoride in the 6 subjects (18) were analyzed, the renal clearance iv averaged 70.2 ± 16.6 ml/min and the renal clearance oral averaged 53.8 ± 16.3 (paired *t*-test for the oral-iv difference yielded a *p* value of 0.007). For 1-deamino-8-arginine vasopressin, the authors explain the high *AUC* ratio based on adsorption of the drug to the syringe following iv dosing (8) and report up to 40% adsorption, which would only partially explain the 66% increase in bioavailability. Additionally, $CL_{R}$ following SubQ is 76% of $CL_{R}$ following iv (the same percentage decrease found in the sodium fluoride study), which is independent of adsorption. Statistical comparison was not possible.

### Justification for Dose-Corrected ${\mathrm{AUC}}_{x}∕{\mathrm{AUC}}_{\mathrm{iv}}>1.0$ and ${\mathrm{AUC}}_{x}∕{\mathrm{AUC}}_{\mathrm{iv}}>U_{\infty x}∕U_{\infty \mathrm{iv}}$

Unless clearance from an absorption site (oral, IM, SubQ) is significantly greater than clearance following an intravenous dose of the drug (Eq. 8 for oral dosing), the *AUC* following absorption dosing may be greater than the *AUC* for an intravenous comparable available dose. When bioavailability is calculated based on systemic concentration measurements, this explains why experimental $AUC_{x}∕AUC_{iv}$ values can often be greater than 1.0 as shown in Table I. On the other hand, when bioavailability is calculated using measures of unchanged drug in the urine, there are no assumptions being made relating absorption processes to elimination processes; therefore, if absorption affects systemic concentrations, $AUC_{x}∕AUC_{iv}$ will be greater than $U_{\infty x}∕U_{\infty iv}$ independent of the F value calculated. A potential challenge to accurately calculating bioavailability using urinary data is the fact that the major route of elimination for Biopharmaceutics Classification System (BCS) and Biopharmaceutical Drug Disposition Classification System (BDDCS) Class 1 and 2 drugs is metabolic elimination (42). Since measures of unchanged drugs will be small, inherent variability will make these comparisons difficult to interpret. As we have reported (42), Class 1 and 2 drugs comprise approximately 70% of all marketed small molecules, making bioavailability measurements using unchanged drugs in the urine questionable.

### Calculation of ${\mathrm{CL}}_{\mathrm{gut}}$ when Both Systemic Concentrations and Urinary Elimination of Unchanged Drug Measures for Oral and iv Dosing Are Available

As indicated in Eq. 8, $CL_{\text{after oral dosing}}$ will be equal to $CL_{iv}$, as is now universally believed and taught, only if $CL_{gut}$ is markedly greater than $CL_{iv}$. Thus, for the data in Table II, it is possible to estimate $CL_{gut}$ and the ratio $CL_{iv}∕CL_{gut}$ if $CL_{iv}$ is available in the publication. Such calculations could be made for all of the studies listed in Table II, except for cilazapril and cilazaprilat. These results are presented in Table III. We demonstrate here this methodology for the cimetidine study (21), where the dose-corrected *AUC* ratio exceeds 1.0 and the urinary and systemic concentration measurements of bioavailability are statistically different. From the published study, the mean values of $U_{\infty \;\text{oral}}∕U_{\infty \;\text{iv}}$, $\frac{{\text{Dose}}_{\text{oral}}}{ACU_{0\to \infty \;\text{oral}}}$ and $CL_{iv}$ were available for the 16 dosings in 9 healthy volunteers as given in columns 2, 3, and 5, respectively, of Table III. Column 4, $CL_{\text{after oral dosing}}$, is the product of the model-independent urinary measure of bioavailability, column 2, and $\frac{Dose_{oral}}{AUC_{0\to \infty \;oral}}$, column 3. Then $CL_{gut}$, column 6, is calculated from the rearrangement of Eq. 8.


(8a) $$CL_{gut}=\frac{CL_{iv}}{\frac{CL_{iv}}{CL_{after\;oral\;dosing}}-1}$$

The last column of Table III then gives the ratio of $CL_{iv}∕CL_{gut}$, which for cimetidine is 1.6. It is unfortunate that this analysis could not be conducted for cilazapril, which showed the greatest statistical difference between the bioavailability measurements using systemic concentrations and urinary bioavailability measurements in Table II, but the reported bioavailability comparisons only used measurements out to 24 h, without providing the potential extrapolated areas (35).

## Discussion

The general approach of our field to the many values presented in Tables I and II is to assume that the measurements are a function of experimental errors, or when the values are divergent but so close to an expected outcome that the divergence is just due to inherent variability. And in many cases, this could be true. In fact, it is often very hard to justify that experimental errors have been made, with the major published justification being that these reported outcomes are not consistent with the present universally accepted pharmacokinetic theory. Examination of the studies for sodium fluoride (18) and cimetidine (21) listed in both Tables I and II reflect the variance found in the literature in discussing the reported results. The data in the crossover studies reported in Table II offer a unique perspective, i.e., the ability to compare paired statistical analyses for two different measurements for the same study. The authors of neither study conducted this analysis, but since individual data for the study subjects were presented in the papers, we were able to conduct this analysis. In the cimetidine study (21), the paired *p* statistic between $AUC_{\text{oral}}∕AUC_{\text{iv}}$ and $U_{\infty \;\text{oral}}∕U_{\infty \;\text{iv}}$ was less than 0.001, while the sodium fluoride study (18) was close, but not statistically different (*p* = 0.055). Both sodium fluoride and cimetidine are BCS/BDDCS class 3 drugs (42) so sufficient amounts of drug in the urine were measurable. The authors of the sodium fluoride study (18) summarized their results in the abstract as “There were large day-to-day variances in renal clearance of fluoride. This was shown to be due to differences in the urinary flow, an increase in flow causing an increase in renal clearance… When apparent bioavailability was calculated from plasma and from urinary data, there was great intra- and intersubject variation, as well as poor agreement between the two methods of calculations. This was found to be due to the day-day variation in renal clearance, which, in turn varied with urinary flow. By use of equations that corrected for these variations, it was found that the bioavailability of sodium fluoride tablets is approximately 100%.” In the sodium fluoride study, there were two overall results that were not consistent with pharmacokinetic theory at the time of the study (and still today). First, mean bioavailability was greater than 100%, although not statistically significant (*p* = 0.055) unless the large difference in one of two studies in subject L.K. was eliminated (*p* = 0.011). Second, $CL_{R,\text{oral}}$ was statistically less than $CL_{R,\text{iv}}$ (*p* = 0.007 paired *t*-test) even when the outlier subject L.K. data were included. The authors therefore needed to explain the reasons that these values cannot be accepted. They proposed that since renal clearance was dependent on urine flow rate, which it is, and renal flow was highly variable, they could ignore the measured oral renal clearances (which are highly significantly different than the measured iv renal clearances) and substitute the iv renal clearances for these values in each subject. Thus, by ignoring the real difference in measured renal clearance following oral dosing, they could show that bioavailability was close to unity. However, renal clearance is renal clearance whether it is highly variable or not and it is difficult to justify why urine flow variability was only observed for the oral dosings, and despite the “high variability,” paired oral and iv renal clearances were highly statistically different (*p* = 0.007). If the authors ignore these measured differences, the reason that F exceeds 1.0 due to absorption affecting systemic concentrations when $CL_{gut}$ is not significantly greater than $CL_{iv\;\text{dose}}$, the measured systemic bioavailability inconsistency will disappear. There is no scientific justification for the conclusion of Ekstrant *et al*. (18) but recognizing that the derivations presented in this present manuscript were unknown until now.

The authors of the cimetidine study (21) did not try to propose an error analysis for their $AUC_{\text{oral}}∕AUC_{\text{iv}}$ equal to 1.106 and $U_{\infty \;\text{oral}}∕U_{\infty \;\text{iv}}$ equal to 0.595 writing “The results clearly demonstrate that bioavailability studies using *AUC* measurements are misleading for several drugs including cimetidine.” However, they did not report the statistical analysis of this comparison (*p* < 0.001 paired test) nor did they calculate or compare $CL_{R}$ oral *vs* iv for their study (*p* = 0.0018 paired *t*-test).

The third significantly different comparison of $AUC_{\text{oral}}∕AUC_{\text{iv}}$ and $U_{\infty \;\text{oral}}∕U_{\infty \;\text{iv}}$ in Table II (*p* < 0.0001) is for active cilazaprilat measurements following cilazapril dosing (35). An important aspect of these data is the recognition that $AUC_{\text{oral}}∕AUC_{\text{iv}}$ measurements may not reflect accurate bioavailability measurements, even when the ratio is less than 1.0. Thus, *AUC* ratio measurements may potentially overestimate actual bioavailability if $CL_{gut}$ is not much greater than $CL_{iv\;\text{dose}}$ (Eq. 8) for drugs of any bioavailability. In therapeutic practice and drug approval of ACE inhibitors, the prodrug is dosed to increase solubility and bioavailability before hydrolysis to its active molecule *in vivo*. The actual amount of active drug that reaches the systemic circulation may be lower than the results predicted using $AUC_{\text{oral}}∕AUC_{\text{iv}}$. This is reflected in the results of the parallel study in the same 12 subjects where cilazaprilat was dosed orally and intravenously (35). Here, following cilazaprat dosing, the ratio of $U_{\infty \;\text{oral}}∕U_{\infty \;\text{iv}}$ to $AUC_{\text{oral}}∕AUC_{\text{iv}}$ for the active drug was 0.64, even lower than the ratio of 0.74 following cilazapril dosing, yet the difference was not significant (*p* = 0.055). This is due to the increased intersubject variability that is observed as measures of bioavailability are decreased as documented by Hellriegel *et al*. (43).

When experimental studies yield systemic bioavailability measures greater than 1.0, investigators, particularly for human studies, try to explain why the results are either inconsequential or due to a confounding experimental error that can be corrected, suspecting that otherwise the study may not be accepted for publication. Since in this manuscript we justify why systemic F>1.0 and $CL_{R,\text{oral}}<CL_{R,\text{iv}}$ results may be obtained and that such results are not necessarily in error, here we summarize explanations of such results for 6 human studies. (a) For 1-deamino-8-arginine vasopressin, the authors (8) try to explain the 1.66 *AUC* ratio based on adsorption of the drug to the syringe following iv dosing, which they reported could be as great as 40%, but not the 66% increase in bioavailability. Furthermore, if absorption has no effect on AUC, $CL_{R}$ should be the same for the iv and SubQ doses independent of adsorption. However, the SubQ $CL_{R}$ is 76% of that following iv dosing. (b) The supposed corrected bioequivalence values for sodium fluoride (18) were obtained by replacing the measured $CL_{R,\text{oral}}$ values in each subject following oral dosing with measured $CL_{R,\text{iv}}$ values believing that $CL_{R}$ should be the same following iv and oral dosing. (c) A paired *t*-test analysis for cimetidine, not previously carried out by the authors (21), shows that the dose-corrected areas following oral dosing are greater than iv with *p* < 0.001. As noted by the authors, a study showing comparable bioavailability was published the previous year by US investigators (44). However, the oral dosage forms were not the same, with a 300 mg tablet manufactured in the US *vs* a 200 mg tablet manufactured in Sweden. (d) The levetiracetam analysis (23) shows that the 90% confidence interval of 105–113% around the 109% mean suggesting that the systemic bioavailability greater than 1.0 is statistically significant. (e) The authors report F for hydroxyurea in 22 patients to be 108 ± 19% (27). No statistics are reported for the bioavailability studies. However, the authors do report a difference in renal clearance oral *vs* iv “with a moderate inverse relationship between the AUC and renal clearance of hydroxyurea (*r* =−0.59, *p* < 0.01)”. (f) Since the mean systemic F value for the ofloxacin study (26) was 1.05, one might suspect that this result being so close to 1.0 may just be due to the normal variance found in human studies. However, the authors report “Because of a small intrasubject variability (coefficient of variation, 4.5%) in the AUC values, the difference in the plasma AUC values between the p.o. and i.v. doses (4.7%) was found to be statistically significant (*p* < 0.05), with the p.o. dosage form having the larger AUC.” Thus, at least for these six human studies, we believe there is compelling evidence supporting that systemic bioavailability can exceed 100% and these results are not attributable to experimental errors.

The common response we receive when documenting the systemic F>1.0 studies is “How do you know that the results are not just the function of saturation of elimination processes for the oral, IM or SubQ doses?” Saturation phenomena related to elimination could be a possible explanation when the oral, IM, or SubQ dose is greater than the iv dose; however, we could not identify any studies where experimental data supported this explanation in the studies listed in Tables I and II. However, there are a number of studies that show that saturation is not the explanation when one examines renal clearance data. For example, in the cimetidine study (21), 9 subjects received a 100 mg iv dose with mean $CL_{R,\text{iv}}=35.6\pm 10.0\;L∕h$, 3 of the 9 received a 100 mg oral dose with mean $CL_{R,\text{oral}}=27.0\pm 10.6\;L∕h$, all 9 received a 400 mg oral dose with mean $CL_{R,\text{oral}}=21.6\pm 10.6\;L∕h$, and 4 received an 800 mg oral dose with mean $C_{R,\text{oral}}=27.2\pm 15.2\;L∕h$. For the sodium fluoride study (18), all 6 subjects received an iv dose of 3 mg with a mean $CL_{R,\text{iv}}=70.2\pm 16.7.6\;\text{ml}∕\text{min}$. Those 6 subjects received a low oral dose (2.82 mg 4 subjects or 4 mg 2 subjects) with a mean $CL_{R,\text{oral}}=55.1\pm 13.0\;\text{ml}∕\text{min}$. Four of the subjects received a higher oral dose (9.4 mg 3 subjects or 5.5 mg 1 subject) with a mean $CL_{R,\text{oral}}=51.8\pm 22.5\;\text{ml}∕\text{min}$. None of the results for cimetidine or sodium fluoride support a saturation effect.

For the remaining 15 human studies listed in Table I, all but desmin were carried out investigating the same dose iv and oral, IM, or SubQ and no indication of any saturation effects is presented in any of the studies. Thus, there are no data or essentially even the possibility that saturation can explain the F>1 values in Table I.

Following our initial presentation of Kirchhoff’s Laws (3), Korzekwa and Nagar (38) questioned our approach. Their position was that Kirchhoff’s Laws could not be used to define the pharmacokinetics of a drug following iv bolus dosing in different multicompartment models. As we explained above, Kirchhoff’s Laws allow the determination of total clearance for rate-defining clearance processes in parallel or in series. There are no individual rate-defining processes following iv bolus dosing. Clearance of multicompartment models (2 and 3 and greater compartments with elimination coming out of the central measured compartment, or from a hypothesized unmeasureable compartment, or from both the measured and unmeasurable compartments) all give the same clearance, i.e., dose eliminated divided by the exposure of drug in the systemic circulation (2). None of the multicompartment distribution and elimination parameters analyzed by Korzekwa and Nagar (38) ever can be considered rate-defining processes. Therefore, the rate-defining processes for all of the models presented by Korzekwa and Nagar are hepatic blood flow, hepatic elimination by metabolism and biliary excretion, and potentially hepatic influx minus efflux, just as would be considered for any iv bolus dose, irrespective of the multicompartment characteristics of the disposition model. We do recognize that the Korzekwa and Nagar paper (38) was written prior to the publication of our second Kirchhoff’s Laws paper (4) where we defined in greater detail the relevance of rate-defining processes. More recently, Rowland *et al*. (39) have also questioned the validity of Kirchhoff’s Laws approach and as we were preparing the response to review for this manuscript, the publication of Professor Siegel (45) has been accepted by this journal. Their papers (as well as the paper of Korzekwa and Nagar) only provide theoretical arguments as to why our approach is not valid, which we will address in the future. Our argument with the Rowland and colleagues and Siegel approaches is that they do not provide information as to how their methodologies explain experimental measurements or address our challenge to provide experimental justification for the different mechanistic models of hepatic elimination (46). We served as a reviewer for the Siegel manuscript, and although we disagreed with the points made, we recommended publication since we are proposing what may be considered a revolutionary revision of some basic pharmacokinetic concepts and it is important for the field to have access to the varying points of view, which can then be addressed based on published theoretical and experimental arguments. In this manuscript, we provide an explanation based on Kirchhoff’s Laws that cannot be explained by differential equation approaches, for why bioavailability measurements based on systemic concentrations may exceed unity, why it is possible to have marked differences in bioavailability based on systemic concentrations *versus* urinary measurements of unchanged drug, why renal clearance measurements following slow-release oral, IM, and SubQ dosing can be less than renal clearance following iv bolus dosing, and as we explained elsewhere (3, 4) why all hepatic elimination data appear to be described best by what we previously believed to be the well-stirred model. In contrast, the Rowland *et al*. and Siegel approaches require one to believe that all of the experimental data exhibiting what the field now considers as anomalous results must result from experimental errors, even in those cases described above where the differences found in these crossover studies are highly statistically significant.

The major objective of this report was to demonstrate that studies resulting in F>1.0 and/or marked differences in systemic *vs* urine predictions in bioavailability may be accurate and should not be considered experimentally flawed. Furthermore, it is incorrect to assume that following oral, IM, and SubQ dosing, the rate of absorption from the dosing site has no effect on the measured area under the curve, as is presently universally believed. However, we recognize that this belief has been the result of having no way to determine the correct relationship between clearance and the rate of absorption, because prior to our introduction of Kirchhoff’s Laws to determine clearance for in-series processes (3, 4) that are inherent in absorption studies, the only derivation of clearance possible was to define the relationship using differential equations in terms of rate constants and then divide by the systemic volume of distribution.

The important question to now address is what effect will these new understandings have with respect to regulatory issues related to bioavailability? Foremost, it must be recognized that the measured *AUCs* following oral, IM, and SubQ dosing are not affected by the analyses reported here. Regulatory guidances for assessing bioequivalence and food effect study data are only based on the measured *AUC* and characteristics of *AUC* related to absorption rate criteria, i.e., $C_{max}$ or *AUC* up to peak time or some chosen time. Similarly, pharmacodynamic outcomes, such as selecting the appropriate dose and dosing interval for a new drug or adjustments in drug dosing due to disease states, drug interactions, or pharmacogenomic and physiologic differences, are only based on *AUC* measurements. So, what will change? First, reported bioavailability values may be an overestimate unless dose-corrected systemic concentration and urinary excretion ratios are similar. Second, since food effect studies often result in changes in bioavailability, can the food effect be a change in gut volume of distribution as well as a change in gut rate of absorption? That is, are there discontinuities between rate constant changes and gut clearance changes that have not been addressed previously? Third, bioavailability studies with F>1.0 or studies with significant differences in ratio for *AUC* and unchanged drug in the urine are no more likely to be experimentally flawed than any other study. Fourth, although the changes described here should have no effect on regulatory issues related to bioavailability and drug dosing decisions, present attempts to predict drug bioavailability, bioequivalence, and food effects using PBPK models may not be considering all relevant aspects of drug absorption. Finally, fifth, if increased dose-corrected *AUC* following slow input into the systemic circulation can be observed, it will be useful for the Regulatory Agencies to recognize that this is not necessarily a saturation effect or an unexplained subject-drug, disease-drug, or drug-drug interaction. Furthermore, the field may find increased pharmacodynamic results for such studies including continuous zeroorder infusion that have not been evaluated previously—a topic we will pursue in future publications.

## Figures and Tables

**Table I:** Published Crossover Studies Where Dose Corrected ${\text{AUC}}_{x}∕{\text{AUC}}_{\text{iv}}$ Is Greater Than 1.02

Drug	Species	Route	${\text{AUC}}_{x}∕{\text{AUC}}_{\text{iv}}$	Reference
Tildipirosin	Horse	SubQ	4.01	(5)
Desmin	Healthy humans	SubQ	1.67	(6)
Tolfenamic acid	Sheep	IM	1.67	(7)
1-Deamino-8-arginine vasopressin^a^	Healthy humans	SubQ	1.66	(8)
Ampicillin	Healthy elderly	SubQ	1.61	(9)
S-Ketorolac	Goat	Oral	1.39	(10)
R-Ketorolac	Goat	Oral	1.36	(10)
Danofloxacin	Chukar partridge	SubQ	1.34	(11)
Ceftazidime	Dog	IM	1.33	(12)
Tolfenamic acid	Sheep	SubQ	1.31	(7)
Enrofloxacin	Lactating cows	SubQ	1.30	(13)
Marbofloxacin	Llama	SubQ	1.27	(14)
Theophylline (tablet)	Dog	Oral	1.26	(15)
Marbofloxacin	Lamb	SubQ	1.25	(16)
Amoxycillin	Dog	IM	1.24	(17)
Sodium fluoride^b^	Healthy humans	Oral	1.23	(18)
Theophylline (capsule)	Dog	Oral	1.23	(15)
Ampicillin	Healthy young	SubQ	1.19	(9)
Treprostinil sodium^c^	Healthy humans	SubQ	1.13	(19)
Tolfenamic acid	Sheep	IM	1.13	(7)
Morphine sulfate	Goat	SubQ	1.11	(20)
Cimetidine^e^	Healthy humans	Oral	1.11	(21)
Teicoplanin^d^	Healthy humans	IM	1.12	(22)
Levetiracetam^f^	Healthy humans	Oral	1.09	(23)
Marbofloxacin	Llama	IM	1.09	(14)
Ketorolac tromethamine	Healthy humans	IM	1.08	(24)
Hydroxyurea^g^	Patients with solid tumors	Oral	1.08	(25)
Fludarabine	Lupus nephritis patients	SubQ	1.07	(26)
Ofloxacin^h^	Healthy humans	Oral	1.05	(27)
Amoxycillin	Dog	SubQ	1.05	(17)
Roquinimex	Healthy humans	Oral	1.04	(28)
Morphine sulfate	Goat	IM	1.04	(11)
Ibuprofen	Healthy humans	Oral	1.03	(29)
Dexmedetomidine	Healthy humans	IM	1.03	(30)

a The authors explain the high AUC ratio based on adsorption of the drug to the syringe following iv dosing, but ${\text{CL}}_{R}$ following SubQ dosing is 76% of ${\text{CL}}_{R}$ following iv, which is independent of adsorption

b *p* = 0.055 for 10 measurements in 6 subjects. When one of the two measurements in subject LK, the dosing involving the greatest change in AUC is deleted; *p* = 0.011 for 9 measurements in 6 subjects. The authors explain the F>1.0 results to be a function of not considering urine flow rates. However, they do not make adjustments based on urine flow rates, but rather replace all measured renal clearances following oral dosing with the renal clearance following iv dosing. When they do this, the AUC ratio decreases to approximately 1.0

c The authors report the standard deviation for the measurements is 1.13 ± 0.10 but provide no statistical analysis

d Both AUC and unchanged urinary excretion ratios exceed 1.0 and appear not to be different

e *p* < 0.001

f The authors report that the 90% confidence interval for the mean 1.09 ratio is 1.05–1.13, suggesting that the mean ratio is statistically significantly greater than 1.0

g The authors reported F in 22 patients to be 108 ± 19%. No statistics are reported for the bioavailability studies. However, the authors do report a difference in renal clearance oral *vs* iv “with a moderate inverse relationship between the AUC and renal clearance of hydroxyurea (*r* = −.59, *p* < .01)”

h *p* < 0.05; 90% confidence interval 1.01–1.08. The authors report: “Because of a small intrasubject variability (coefficient of variation, 4.5%) in the AUC values, the difference in the plasma AUC values between the p.o. and i.v. doses (4.7%) was found to be statistically significant (*p* < 0.05), with the p.o. dosage form having the larger AUC.”

**Table II:** Published Crossover Studies in Healthy Humans Exhibiting Differences in Oral Bioavailability Using Dose Corrected AUC (${\text{AUC}}_{\text{oral}}∕{\text{AUC}}_{\text{iv}}$) *Versus* Urinary Excretion ($U_{\infty \;\text{oral}}∕U_{\infty \;\text{iv}}$) Data

Drug	${\text{AUC}}_{\text{oral}}∕{\text{AUC}}_{\text{iv}}$	$U_{\infty \;\text{oral}}∕U_{\infty \;\text{iv}}$	Reference
Sodium fluoride	1.233 ± 0.308	0.886 ± 0.191^a^	(18)
Cimetidine	1.119 ± 0.399	0.595 ± 0.156^b^	(21)
Indoprofen (100 mg capsule)	1.018 ± 0.353	0.891 ± 0.261^c^	(31)
Letrozole	0.991	0.937	(32)
Allopurinol	0.904	0.814	(33)
Indoprofen (200 mg tablet)	0.867 ± 0.146	0.809 ± 0.214^d^	(31)
Hydroxychloroquine	0.79 ± 0.12	0.69 ± 0.15^e^	(34)
Cilazapril (measure cilazaprilat)	0.775 ± 0.101	0.571 ± 0.103^f^	(35)
Mesna	0.680 ± 0.413	0.580 ± 0.173 ^g^	(36)
Ranitidine	0.52 ± 0.11	0.38	(37)
Cilazaprilat	0.290 ± 0.148	0.186 ± 0.099 ^h^	(35)

a *p* = 0.055; 10 paired measurements in 6 subjects; *p* = 0.011 when one of the two paired measurements in subject L.K. not included in the analysis

b *p* < 0.001; 16 paired measurements in 9 subjects

c *p* = 0.411; 4 paired measurements

d *p* = 0.689; 4 paired measurements

e *p* = 0.43; 5 paired measurements

f *p* < 0.0001; 12 measurements of active cilazaprilat dosing cilazapril in 12 subjects. Calculated from means and SDs since individual data not given

g *p* = 0.69; 5 paired measurements

h *p* = 0.055; 12 measurements of cilazaprilat dosing cilazaprilat in the same 12 subjects who were also dosed cilazapril. Calculated from means and SDs since individual data not given

**Table III:** Calculation of Clearance Gut ($CL_{gut}$) and the Ratio of Clearance Gut to Clearance iv for Drugs in Table II Where Bioavailability Measures Using Systemic Concentrations and Unchanged Amounts in the Urine Are Available^a^

Drug	$F=\frac{U_{\infty \;oral}}{U_{\infty \;iv}}$	$\frac{{\text{Dose}}_{\text{oral}}}{AUC_{0\to \infty \;\text{oral}}}$	$CL_{\text{after oral dosing}}$	$CL_{iv}$	$CL_{gut}$	$\frac{{\text{CL}}_{\text{gut}}}{{\text{CL}}_{\text{iv}}}$
		L/h	L/h	L/h	L/h	
Sodium fluoride (18)	0.886	9.81	8.68	10.8	44.3	4.1
Cimetidine (21)	0.595	50.4	30.0	48.5	78.6	1.6
Indoprofen (capsule) (31)	0.891	3.48	3.10	3.47	29.1	8.4
Letrozole (32)	0.937	2.04	1.91	2.21	14.1	6.4
Allopurinol (33)	0.814	50.7	41.3	46.6	363	7.8
Indoprofen (tablet) (31)	0.867	3.39	2.94	2.97	291	98
Hydroxychloroquine (34)	0.69	63.9	44.1	50.0	374	7.5
Mesna (36)	0.58	123	71.5	77.9	870	11
Ranitidine (37)	0.38	84.7	32.2	44.6	116	2.6

a Values in the table are calculated from urinary excretion bioavailability (F) measurements since no assumptions related to the effect of input on the measured *AUC* values are inherent in the urinary values; $\frac{{\mathrm{Dose}}_{\mathrm{oral}}}{{\mathrm{AUC}}_{0\to \infty \;\mathrm{oral}}}$ is the stated amount of the oral dose divided by the measured *AUC* 0 to ∞ reported; $CL_{\text{after oral dosing}}$ is calculated by Eq. 8, the product of the values in the two previous columns; $CL_{iv}$ is the value reported in the publication following iv bolus dosing; knowing $CL_{\text{after oral dosing}}$ and $CL_{iv}$, then $CL_{gut}$ is calculated from the values in the two previous columns using a rearrangement of Eq. 8; the ratio in the last column is calculated to characterize whether or not input clearance ($CL_{gut}$) has an effect on measured clearance following oral dosing ($CL_{\text{after oral dosing}}$), outlining which studies result in values that are not consistent with the universally accepted hypothesis that input has no effect on the measured clearance following oral dosing. That is, the low ratios for cimetidine, ranitidine, and sodium fluoride suggest that for these studies, conventional pharmacokinetic theory may not be correct. In contrast for the remaining drugs, within experimental error, the clearance measured following the oral dose will be the same as that following the iv bolus dose

## Data Availability

All data generated or analyzed during this study are included in this article or in the references provided.

## Abbreviations

$A_{\text{systemic cirulation}}$: Amount of drug in the systemic fluids
ACE: Angiotensin converting enzyme
AUC: Area under the systemic concentration-time curve
BCS: Biopharmaceutics Classification System
BDDCS: Biopharmaceutics Drug Disposition Classification System
$C_{\max}$: Maximum systemic drug concentration
$C_{\text{systemic cirulation}}$: Concentration of drug in the systemic circulation
CL: Clearance
${\text{CL}}_{\text{gut}}$: Clearance of drug from the gut following oral dosing
${\text{CL}}_{H}$: Hepatic blood clearance
${\text{CL}}_{R}$: Renal blood clearance
F: Bioavailability
F(AUC): Bioavailability calculated from systemic concentrations
F(urine): Bioavailability calculated from urinary excretion amounts
IM: Intramuscular
iv: Intravenous
k: Rate constant
$k_{a}$: Absorption rate constant
$k_{d}$: Elimination rate constant for a one-compartment body model
$k_{\text{ss}}$: Overall elimination rate constant at steady-state for multicompartment body models
MAT: Mean absorption time
MRT: Mean residence time
$Q_{H}$: Hepatic blood flow
SubQ: Subcutaneous
$U_{\infty }$: Amount of drug in the urine at infinite time
V: Systemic volume of distribution
x: Subscript reflecting parameter for either oral, intramuscular, or subcutaneous dosing
